# Development of chronic subdural haematoma from mild head injury: A case report and review of current Malaysian guidelines on traumatic brain injury

**DOI:** 10.51866/cr.776

**Published:** 2025-07-12

**Authors:** Qingping Joseph Feng, Ian James Long, Su Lone Lim, Ira Siyang Sun, Shiong Wen Low, Chun Peng Goh

**Affiliations:** 1 MBBS, MRCS, Department of Neurosurgery, Division of Neurosurgery, Department of General Surgery Ng Teng Fong General Hospital, 1 Jurong East Street 21, Singapore. E-mail: joseph.feng@mohh.com.sg; 2 MBBch BAO, Division of Neurosurgery, Department of General Surgery Ng Teng Fong General Hospital, 1 Jurong East Street 21, Singapore.; 3 MD, MRCS, FRCS (Neurosurgery), Division of Neurosurgery, Department of General Surgery Ng Teng Fong General Hospital, 1 Jurong East Street 21, Singapore.; 4 MBBS, MRCS, FRCS (SN), MS HPEd, Division of Neurosurgery, Department of General Surgery Ng Teng Fong General Hospital, 1 Jurong East Street 21, Singapore.; 5 MBBS, MRCS, MMed (Surgery), FRCS (Neurosurgery), FAMS (Neurosurgery), LLB (Hons), Division of Neurosurgery, Department of General Surgery Ng Teng Fong General Hospital, 1 Jurong East Street 21, Singapore.; 6 MBBS, MRCS, MCI, FRCS (SN), Division of Neurosurgery, Department of General Surgery Ng Teng Fong General Hospital, 1 Jurong East Street 21, Singapore.

**Keywords:** Brain injuries, Post-traumatic complications, Subdural haematoma, Delayed diagnosis, Primary health care

## Abstract

Delayed chronic subdural haematoma (cSDH) is a common but potentially serious complication following traumatic brain injury (TBI). Mild TBIs are commonly managed by primary care providers (PCPs), particularly in large, resource-limited settings such as Malaysia, where access to tertiary neurosurgical services may be delayed. Early identification of red-flag signs and symptoms and timely referrals are crucial to prevent clinical deterioration. We describe the case of a 66-year-old man who sustained mild head injury following a vasovagal syncope. His initial brain CT revealed evidence of a small traumatic subarachnoid haemorrhage over the left precentral sulcus, with resolution on an interval scan 24 hours later. He was discharged home without follow-up. Eleven weeks later, he developed bilateral lower-limb weakness and unsteady gait, which prompted an urgent referral by his general practitioner. Repeat CT revealed bilateral acute-on-chronic subdural haematomas, with mass effect requiring emergency burr-hole drainage. The patient showed excellent post-operative improvement and was discharged home on day 4, with no clinical or radiological recurrence on subsequent follow-up. This case highlights the risk of delayed cSDH in patients following mild TBI, even in those discharged with a normal CT scan. PCPs play a pivotal role in recognising high-risk patients, ensuring structured follow-up and facilitating timely specialist referral. We advocate for updating the Malaysian head injury guidelines to incorporate routine follow-up protocols for at-risk patients, modelled after international standards.

## Introduction

Delayed chronic subdural haematoma (cSDH) is a well-recognised yet underdiagnosed complication following traumatic brain injury (TBI). A 2018 study estimated that approximately 69 million individuals globally experience TBI annually, with Southeast Asian and African countries bearing the greatest overall burden.^1^ The majority of TBIs (81%) are categorised as mild^1^ and are often managed in primary care settings or emergency departments (EDs) without specialist involvement.

Malaysia faces unique challenges in TBI management due to geographic disparities, resource limitations and the high prevalence of road traffic accidents that account for 64% of TBIs in the country.^2,3^ Access to tertiary neurosurgical centres is often delayed, and primary care providers (PCPs) play a crucial role in early assessment, management and follow-up care.

This report presents a case of symptomatic cSDH following mild TBI with a normal interval brain CT scan. We explore the role of PCPs in managing at-risk patients, review current local guidelines and propose updates to prevent missed delayed complications.

## Case presentation

A 66-year-old man presented to our institution’s ED with a 2-day history of epigastric discomfort. His medical history was significant for asthma, pangastritis, chronic rhinosinusitis and atypical chest pain. He was a nonsmoker and was not on any antiplatelets or anticoagulants.

**Figure 1:**
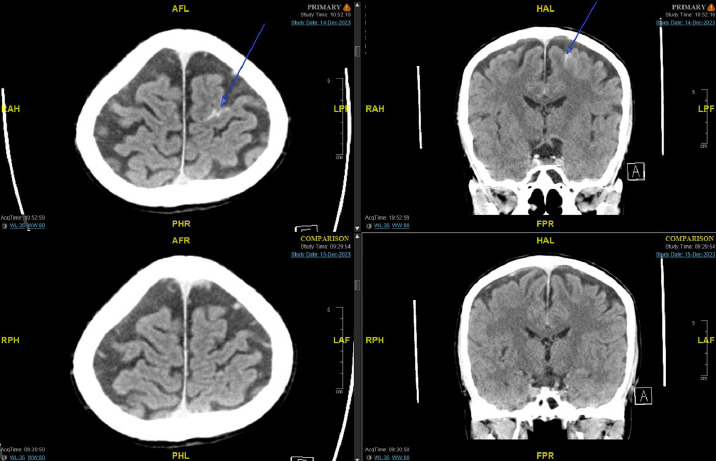
**Top row** - axial (left) cut of index brain CT; coronal (right) cut of index brain CT Arrow(s) highlighting acute subarachnoid haemorrhage (SAH) in the left precentral sulcus. **Bottom row** - axial (left) cut of interval brain CT conducted 22 hours after initial CT, coronal (right) cut of interval brain (CT. These images demonstrate the resolution of SAH.

The patient experienced a syncopial episode during his inpatient stay where he fell forward and sustained a head injury. He exhibited retrograde amnesia but did not report headaches or dizziness. Physical examination recorded a full Glasgow Coma Scale score of 15, bilaterally reactive pupils end no limb weakness. He was normotensive throughout, with the highest blood pressure reading of 124/63 mmHg. A CT scan of the brain revealed *a* small acute subarachnoid haemorrhage (SAH) within the left precentral sulcus (Figure 1, top raw). The patient’s platelet count and coagulation profile were within normal values.

The neurosurgical team was consulted and corroborated the clinical findings. (Conservative management with close neuromonitoring and a 24-hour interval scan was advised. Interval brain CT conducted approximately 22 hours after the initial scan showed resolution of the SAH and no new acute findings (Figure 1, bottom row). The patient remained neurologically intact throughout his inpatient stay. His condition was reviewed by the inpatient physiotherapy and occupational therapy team, deemed to be at his baseline function with no neurological deficit and cleared for discharge home. Verbal head injury advice was given, and there was no routine follow-up arranged.

After discharge, the patient was followed up by multiple specialists at different institutions over the next 10 weeks for his chronic conditions. There was no neurological deterioration or interval development of delayed symptoms recorded in these visits. However, on his fourth visit for gastritis, the patient reported non-specific light-headedness and a ‘floating sensation’ while walking, with subjective lower-limb weakness. Objective testing of his limb power was unremarkable, and he was discharged home with no further neurological investigations conducted. There was no record of acknowledgement on the diagnosis of his previous head injury.

**Figure 2:**
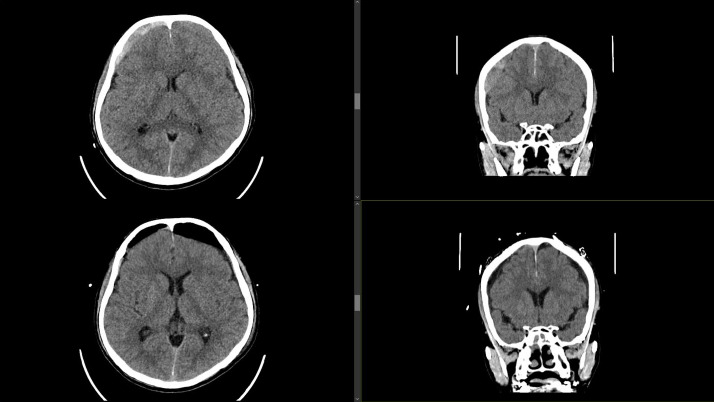
**Top row** - axial (left) and coronal (right) cuts demonstrating bilateral acute-on-chronic subdural haematomas (cSDH). **Bottom row** - axial (left) and coronal (right) cuts demonstrating post-operative burr-hole drainage of cSDH.

The patient presented approximately 1 week later to his general practitioner with a 1-week history of worsening subjective lower-limb weakness and unsteady gait, with no worsening neurology or function. This prompted an urgent referral to the closest neurosurgical unit for further evaluation. On review, there was no complaint of headaches, constitutional symptoms, upper-limb weakness or numbness. Objective testing recorded full limb power in all four limbs. Repeat brain CT showed bilateral acute-on-chronic subdural haematomas with effacement of the underlying brain parenchyma (Figure 2, top row).

**Figure 3:**
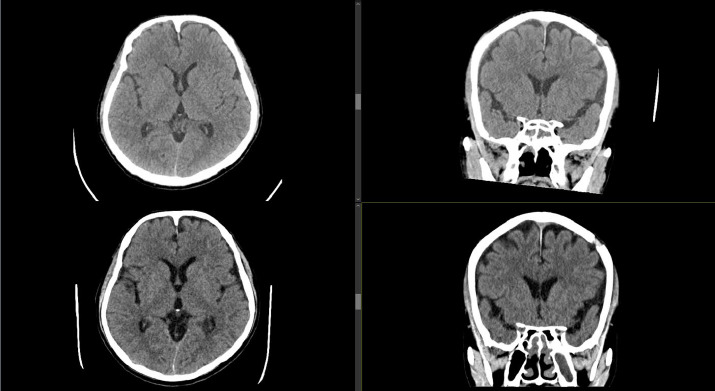
**Top row** - axial (left) and coronal (right) cuts demonstrating no recurrence at 1 month after surgery. **Bottom row** - axial (left) and coronal (right) cuts demonstrating no recurrence at 6 months after surgery.

The patient underwent an emergency burr-dole drainage of his bilateral subdural haemattomas with symptomatic improvement. Post-operative CT (Figure 2, bottom row) performed 2 days after surgery showed a reduction in the size of the subdural collections. He recovered well and was subsequently discharged on post-operative day 4. Follow-up CT conducted at 1 and 6 months after surgery (Figure 3) did not show any recurrence.

## Discussion

Mild TBI accounts for the majority of head injuries worldwide. However, there remains no clear consensus on the optimal follow-up strategy for detecting delayed complications such as cSDH. This case highlights the critical role of PCPs in identifying at-risk patients and underscores the need for structured follow-up protocols. Herein, we discuss the pathophysiology of delayed cSDH, review local Malaysian and international guidelines and propose recommendations to enhance post-TBI care in Malaysia.

### Pathophysiology of delayed cSDH development after mild TBI

cSDH typically results from trauma-induced rupture of bridging veins that traverse the subdural space between the cerebral cortex and dural venous sinuses.^4,5^ The initial injury may cause a small, self-limiting bleed, often undetectable on early neuroimaging. However, subsequent inflammatory and angiogenic processes from fragile neovascularised membranes contribute to haematoma expansion over time.^4^ Along with hyperosmolar fluid shifts, this leads to increasing mass effect, cortical compression and progressive neurological decline. These mechanisms explain the development of cSDH anytime from weeks to months, necessitating long-term vigilance, especially in high-risk individuals.

### Review and comparative analysis of local Malaysian guidelines

The Malaysian Health Technology Assessment Section (MaHTAS) published a clinical practice guideline (CPG) for early management of head injury for adults in 2015.^6^ While the guideline provides clear discharge criteria and risk stratification tools, it lacks specific recommendations for routine follow-up patients with an initially normal CT scan, potentially increasing the risk of missed delayed complications. The guideline may also be outdated, with its last revision in 2015.

In contrast, the United Kingdom’s National Institute of Health and Care Excellence (NICE) guidelines^7^ are regularly reviewed with integration of new evidence into practice. There is an emphasis for a structured follow-up programme for high-risk patients, including scheduled post-discharge assessments for those with persistent and/or post-concussion symptoms and/or with high-risk factors such as older age and anticoagulation use. Additionally, the NICE guidelines acknowledge the potential for late deterioration risk, supports the role of PCPs in post-TBI monitoring and facilitates early re-evaluation and specialist referral when indicated.

Given the increasing global burden of cSDH, particularly in ageing populations, the absence of structured follow-up in the MaHTAS guidelines may result in the underdiagnosis or late detection of cSDH cases, resulting in poorer patient outcomes. To address this gap, we propose the following changes modelled after best international practices.

### Proposed changes to the MaHTAS guidelines

**1. Implementation of structured follow-up**

Delayed cSDH can manifest weeks to months post-injury.^4,5^ The prospective cohort study by Karibe et al.^8^ detected cSDH in 19.9% of older adult patients within 4-12 weeks following mild TBI. Similarly, multiple retrospective analyses also reported that a significant proportion of patients who had cSDH presented with neurological deterioration within 30–60 days from initial injury.^9,10^

Based on the findings, we recommend structured follow-up within the first month of injury to enable early detection of delayed complications, particularly in highrisk patients; this suggestion balances the detection of complications along with the practical considerations of patient recovery and resource availability.

**Figure 4:**
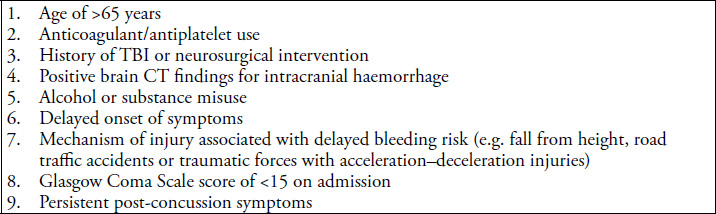
Characteristics of patients at a high risk of delayed deterioration.^6,7^

The following patient characteristics (Figure 4) should guide clinicians in identifying patients who warrant close monitoring:

**Recommendation: A 1-month post-injury follow-up should be mandatory for high-risk patients, with a clear protocol for escalation of care if deterioration occurs.**

**2. Primary care physician led-monitoring**

Patients with mild TBI and normal neurological findings can often be safely monitored in the community. The prospective analysis by Sifri et al.^11^ concluded that routine interval CT scans for patients with TBI and normal neurological findings did not affect management. The incidence of cSDH requiring surgical intervention was reported to be 0.13% by Heinonen et al.,^12^ supporting the selective use of neuroimaging.

Nevertheless, PCPs should be empowered to directly refer patients to neurosurgical centres for further management if there are clinical signs of deterioration. A standardised post-TBI assessment tool should be introduced to aid primary care physicians in identifying patients requiring urgent neuroimaging and neurosurgical review.

**Recommendation: Establish a standardised PCP-led assessment tool and direct referral system to neurosurgical centres for suspected cases of delayed cSDH.**

**3. Enhancing awareness of the delayed complications of mild TBI**

One of the key contributors to late diagnosis is insufficient patient and caregiver awareness about delayed cSDH symptoms. The current discharge protocol lacks explicit warnings about potential late deterioration, leading to delayed medical attention when symptoms arise.

Discharge advice should be reviewed to include specific warnings about delayed neurological symptoms and emphasise the need for prompt reassessment if deterioration occurs. Additionally, written and verbal education for patients and caregivers should be standardised across all healthcare facilities.

**Recommendation: Standardise written and verbal patient education on delayed cSDH symptoms across healthcare facilities to ensure early symptom recognition and timely medical intervention.**

## Conclusion

This case highlights the risk of delayed cSDH in patients with mild TBI, even when the initial CT scan shows resolution of the previous head injury. PCPs play a pivotal role in recognising high-risk patients and ensuring appropriate follow-up. We advocate for an update to the Malaysian CPG to incorporate structured follow-up in mild TBI cases to improve early detection and enhance patient safety.
